# Temporomandibular joint dislocation in patients with cleft lip and palate after maxillary distraction osteogenesis: Three case reports

**DOI:** 10.1097/MD.0000000000024012

**Published:** 2021-02-12

**Authors:** Binqing Wang, Junya Zhai, Yilue Zheng, Haizhou Tong, Yang Lü, Zhewei Chen, Ningbei Yin, Tao Song

**Affiliations:** a Center for Cleft Lip and Palate Treatment; b Center of Maxillofacial Surgery and Digital Plastic Surgery, Plastic Surgery Hospital, Chinese Academy of Medical Sciences and Peking Union Medical College, Beijing, China.

**Keywords:** cleft palate, maxillary distraction osteogenesis, maxillofacial malformation, temporomandibular joint dislocation

## Abstract

**Introduction:**

Distraction osteogenesis (DO) is a widely used for cleft and palate related maxillary hypoplasia. There has been little research on temporomandibular joint (TMJ) dislocation after maxillary DO. We present these 3 cases and analyze the possible causes for reference by other clinicians.

**Patient concerns:**

In the late stages of maxillary DO, the patients gradually felt a decrease in mandibular mobility and suffered from limited mouth opening. Case 2 and 3 could open their mouth up to 1 and 2 fingers and Case 1 barely able to open her mouth at the completion of distraction.

**Diagnosis:**

Case 1 and Case 3 were diagnosed as right TMJ dislocation and Case 2 had a TMJ dislocation on her left side.

**Interventions:**

Patients with TMJ dislocation were repositioned with manipulation as soon as detected.

**Outcomes:**

There was no recurrence in all three cases during the postoperative follow-up period.

**Conclusions:**

Maxillary DO can sufficiently advance the maxilla in cleft lip and palate patients. Clinicians should be mindful of the TMJ dislocations that maxillary DO can exert on patients.

## 1 Introduction

Maxillary deficiency is a common secondary deformity associated with cleft lip and palate (CLP). A considerable number of patients with Angle class III malocclusion need orthognathic surgery in either adolescence of adulthood. Since the rigid external distraction (RED) device was introduced for the management of severe midface hypoplasia by Polley and Figueroa in 1997,^[1]^ classic Le Fort I osteotomy followed by maxillary distraction osteogenesis (DO) has been widely used for patients with CLP to advance their hypoplastic maxilla. This type of technology has been shown to sufficiently advance the maxilla and reduce relapse regardless of whether the patient is an adolescent or adult.^[2,3]^ Our center has made some modifications to the RED device by using bone-borne intranasal traction in order to avoid dentoalveolar compensation during traction.^[4]^ However, in our clinical practice, we encountered three patients who experienced temporomandibular joint (TMJ) dislocation and limited mouth opening after maxillary DO. To the best of our knowledge, there has been little research on this topic. Here, we describe these three cases.

## 2 Methods

### 2.1 Ethics

The Plastic Surgery Hospital, Chinese Academy of Medical Sciences, and Peking Union Medical College ethics committee approved this study, which was performed according to the principles of the Declaration of Helsinki. Informed written consent was obtained from the patients for publication of this case report and accompanying images.

### 2.2 Surgical technique

During surgery, we first performed a classic Le Fort I osteotomy; then, a RED device was placed. The anchor points were located 1 cm outside the lateral pyriform rims and 5 mm above the apices of the teeth. Distraction began immediately after osteotomy, with an initial force of 250 g on each side and a direction of 20° off the Frankfort horizontal plane. After a latency period of 3 to 4 days, distraction was initiated with a variable rate of 1 to 2 mm each day during the changing of springs. The maxilla moved slowly with continuous elastic distraction. The distraction usually requires 4 weeks. The end of the distraction was determined by an occlusal analysis and cephalometry. Then, the RED frame and traction hooks were removed and a maxillary fixation was performed with L-shaped titanium plates (thickness, 1.0 mm) and 4 screws (diameter, 2.0 mm; length, 6.0 mm).

### 2.3 Radiographic analysis

Three-dimensional computed tomography (CT) evaluations were performed before surgery and after distraction.

**Figure 1:**
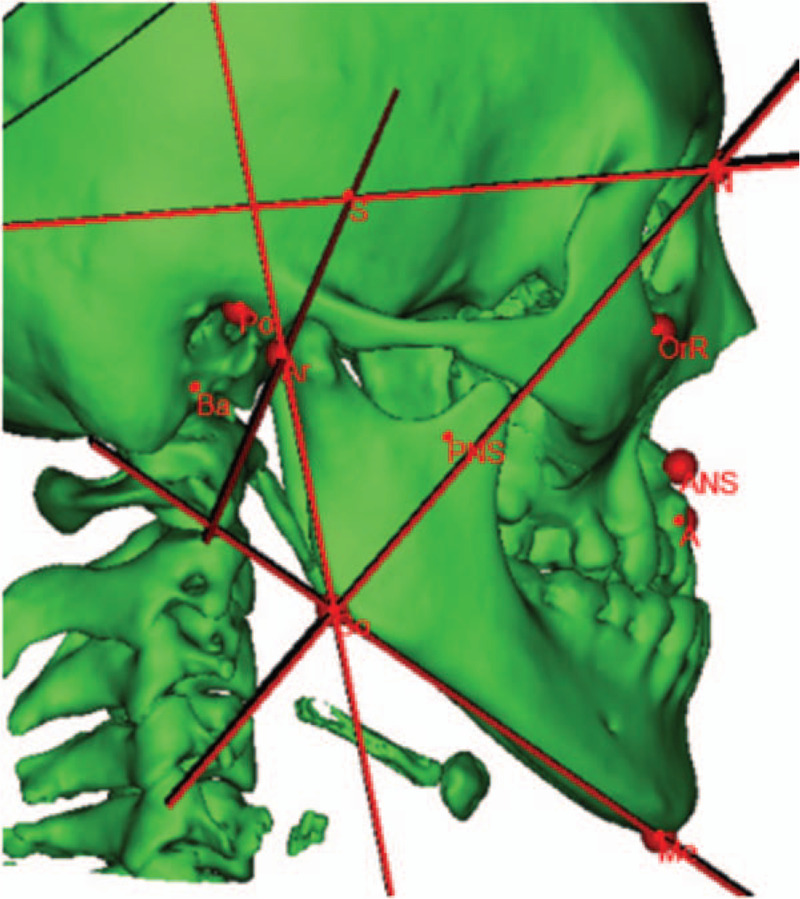
Bony landmarks used in this analysis. A = subspinale, ANS = anterior nasal spine, Ar = articulare, Ba = basion, Go = gonion, Me = menton, N = nasion, Or = orbitale, PNS = posterior nasal spine, Po = porion, S = sella.

The bony landmarks used for analysis included the sella (S), nasion, subspinale, anterior nasal spine, posterior nasal spine, menton, gonion (Go), articulare, basion (Ba), porion, and orbitale. The sagittal plane was determined by the S, N, and Ba points. The following planes were determined by 2 points and were perpendicular to the sagittal plane (Fig. 1): Frankfort horizontal plane (FH/Or- porion); palatal plane (PP/anterior nasal spine - posterior nasal spine); sella-articulare plane (SAr/S-Ar); articulare-gonion plane (ArGo/Ar-Go); nasion-gonion plane (NGo/N-Go); mandibular plane (MP/Me-Go); and sella-nasion plane (SN/S-N). Then, a coronal plane (CP) was determined perpendicular to both the FH plane and sagittal plane through Ba point.

The angular measurements (°) used in the analysis were determined using the following angles between 2 planes: palatal plane angle (PP-FH); mandibular plane angle (MP-FH); saddle angle (SN-SAr); articular angle (SAr-ArGo); upper gonial angle (ArGo-NGo); lower gonial angle (NGo-MP); and gonial angle (ArGo-MP). The following linear parameters were measured between the points cast on the sagittal plane: posterior cranial base (S-Ar); ramus height (Ar-Go); anterior facial height (N-Me); and posterior facial height (S-Go). The distances from the A point to the FH plane and to the CP plane were measured. Additionally, the Jarabak facial proportion (%) was measured using the ratio of the posterior and anterior facial heights.

## 3 Case presentation

### 3.1 Case 1

**Figure 2:**
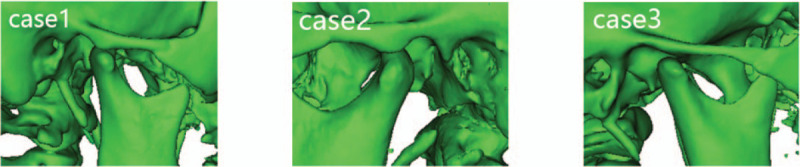
Three-dimensional computed tomography reconstructions of the 3 patients’ temporomandibular joint dislocations.

A 19-year-old female patient was diagnosed with right-sided complete cleft lip and palate, with no family history of cleft lip and palate. She had undergone cheiloplasty and palatoplasty at age 3 months and 12 months. She also underwent palatal fistula repair and secondary cheiloplasty at 8 years old. The patient presented a severe cleft maxillary hypoplasia and malocclusion after puberty. After a Le Fort I osteotomy in July 2017, the distraction was started and lasted for 4 weeks. The A point was moved 25.52 mm anteriorly and 3.37 mm inferiorly after the distraction. In the late stage of distraction, the patient gradually experienced a decrease in mandibular mobility and in mouth opening without significant pain. At the completion of the distraction the patient could barely move the jaws. CT revealed displacement of the right mandibular condyle away from the articular fossa to the anterior inferior aspect of the articular tuberosity (Fig. 2). The measurements revealed a 9.75° decrease in saddle angle and an 11.25° increase in articular angle compared to the preoperative period. After distraction, the palatal plane angle increased by 19.70°, the mandibular plane angle increased by 1.11°, the upper gonial angle decreased 2.64°, and a 4.44° increase in the lower gonial angle was seen, indicating an anterior and inferior displacement and a clockwise rotation of both the maxilla and mandible. The dislocated condyle was immediately repositioned by manipulation. Subsequently, the RED device was removed, and internal rigid fixation of the maxilla, and sagittal split retraction of the mandible were performed. The patient could open her mouth to approximately the width of 2 fingers during the operation. Joint X-rays were normal 1 week after the operation. The patient's mouth opening returned to approximately the width of 3 fingers with no recurrence of joint dislocation during the 1-year postoperative follow-up period.

### 3.2 Case 2

An 18-year-old female patient was diagnosed with right-sided complete cleft lip and palate, with no family history of cleft lip and palate. She had undergone cheiloplasty and palatoplasty at age 3 months and 12 months. In order to improve the facial contour and correct the malocclusion, Le Fort I distraction osteogenesis was performed at our center in July 2019. The distraction lasted for 4 weeks. The A point was moved 13.78 mm anteriorly and 8.56 mm inferiorly after the distraction. Late in the distraction period, the patient progressively experienced a decrease in mandibular mobility. She could open her mouth to about 1 finger without significant joint pain or clicking. CT revealed the left mandibular condyle moving to the inferior aspect of the articular tuberosity (Fig. 2). The measurements revealed a 7.74° decrease in saddle angle and a 10.23° increase in articular angle compared to the preoperative period. After distraction, the palatal plane angle increased by 9.30°, the mandibular plane angle increased by 1.93°, the upper gonial angle decreased 5.00°, and a 6.05° increase in the lower gonial angle was observed, indicating an anterior and inferior displacement and a clockwise rotation of both the maxilla and mandible. The dislocated condyle was immediately repositioned by manipulation. The RED device was then removed and internal rigid fixation of the maxilla performed. The patient could open her mouth to approximately the width of 2 fingers during the operation. The patient returned the degree of mouth opening to approximately the width of 3 fingers during the postoperative follow-up and there was no recurrence of joint dislocation.

### 3.3 Case 3

A 12-year-old male patient was diagnosed with bilateral complete cleft lip and palate, with no family history of cleft lip and palate. He had undergone cheiloplasty and palatoplasty at age 3 months and 12 months. Although the patient had not reached the age of maxillary maturity, bilateral cleft palates resulted in severe maxillary hypoplasia, malocclusion and dental crowding. He had poor pneumatization of the maxillary sinuses. After a Le Fort I osteotomy in October 2018, the distraction was started and lasted for 4 weeks. The A point was moved 14.52 mm anteriorly and 4.60 mm inferiorly after the distraction. Late in the distraction period, the patient's mandibular mobility decreased slightly and mouth opening was reduced to approximately the width of 2 fingers without significant pain. At the completion of distraction, CT showed that the patient's right mandibular condyle was displaced to the anterior inferior aspect of the articular tuberosity (Fig. 2). The measurements revealed a 10.71° decrease in saddle angle and a 14.57° increase in articular angle compared to the preoperative period. It was also found that after distraction, the palatal plane angle increased by 2.75°, the mandibular plane angle increased by 7.69°, the upper gonial angle decreased 6.17°, and a 9.70° increase in the lower gonial angle was observed, indicating an anterior and inferior displacement and a clockwise rotation of both the maxilla and mandible. The dislocated condyle was immediately repositioned by manipulation. The RED device was then removed and internal rigid fixation of the maxilla performed. The patient gradually recovered the degree of mouth opening during the postoperative follow-up and there was no recurrence of joint dislocation.

## 4 Discussion

TMJ dislocation occurs when the condylar process moves in front of the articular eminence and is unable to descend back to its normal position. It can be partial (subluxation) or complete (luxation), bilateral or unilateral, and acute or chronic.^[6]^ The most common is anterior dislocation, as reported here. There have been some reports of TMJ disorders after orthognathic surgery, mainly concerning neuromuscular changes and condylar atrophy related to TMJ dysfunction. Existing studies of the effects of Le Fort I osteotomies on the TMJ are scarce and contradictory. O’Ryan and Epker found a low incidence of postsurgical pathologic masticatory muscle spasms and TMJ dysfunction compared to normal individuals who have no surgical history.^[7]^ Cortez and Passeri indicated that Le Fort I osteotomy for maxillary advancement did not cause any significant changes in the position of the mandibular condyle.^[8]^ Al-Riyami et al performed a systematic review and concluded that patients who underwent bilateral sagittal split osteotomy advancement procedures and LeFort I maxillary impaction procedures are more likely to experience improvements in their TMJ signs and symptoms.^[9]^ However, Kahnberg found TMJ problems such as pain and reduced opening capacity in 60% of the Le Fort I osteotomy cases studied.^[10]^ After the advent of maxillary DO, slow distraction of bone and the histogenic abilities of DO may have few instantaneous influences on the craniofacial muscle.^[11]^ Nevertheless, the impact of DO on the TMJ cannot be generalized because the anterior movement of the maxilla with DO is often larger than the cases with classic Le Fort I osteotomy. Reports related to TMJ problems are rare. Hashimoto et al. suggested that maxillary DO had no effect on the TMJ function of subjects with CLP for up to 1 year after DO.^[12]^ To the best of our knowledge, no studies on TMJ dislocation after either Le Fort I osteotomy or maxillary DO have previously been reported.

This type of postoperative TMJ dislocation could result from 2 possible causes, the effect of maxillary DO and congenital maxillofacial malformation of the CLP. First, it might be associated with changes in the position of the mandible during the traction. Considering that patients who underwent maxillary DO have severe three dimensional hypoplasia of the maxilla, we hypothesized that the mandibular position might be contra-rotated in CLP when compared to that in the Class I malocclusion group. The anterior-inferior movement of the maxilla could induce clockwise rotation of the mandible to fit the new position of maxilla, with this rotation being likely to have an influence on the temporomandibular joint. When the condyle cannot adapt to the change, it may slip to the front of the articular tuberosity Second, congenital craniofacial malformations were found in our three patients according to our analysis. We conducted a Jarabak analysis and compared some parameters using the Jarabak reference values^[5]^ (Table 1). Their cranial base (both anterior and posterior) and posterior facial height were significantly less than normal values (beyond 1 standard deviation), which might be related to the three dimensional hypoplasia of the maxilla in patients with CLP. Additionally, the gonial angles, especially the lower gonial angles in the three patients, were larger than the reference values. According to the Jarabak analysis,^[5]^ the upper gonial angle indicates the inclination of the mandibular ramus, which has a positive correlation with the anterior growing tendency of the chin. The lower gonial angle indicates the inclination of the mandibular corpus. A large lower gonial angle indicates clockwise rotation of the mandibular corpus. In our study, 2 patients had clockwise growth (54%-58%) of the mandible and the other had normal growth (59%-63%) according to the Jarabak ratio (S-Go/N-Me). This indicated that there was a certain degree of clockwise rotation of the mandibular structure before DO. Therefore, even a small rotation of the mandible during maxillary DO could result in dislocation of the condyle. Other possible causes of TMJ dislocation could be neuromuscular changes during distraction and condyle defects^[13]^ in patients with CLP.

**Table 1:** Comparisons of preoperational measurements from the Jarabak analysis.

	Case 1	Case 2	Case 3	Jarabak norms Mean ± SD
Saddle angle (°)	141.09	117.86	145.97	123 ± 6
Articular angle (°)	122.49	145.43	112.29	143 ± 5
Upper gonial angle (°)	54.03	52.16	65.38	52 ± 5
Lower gonial angle (°)	80.31	84.08	86.47	76 ± 3
Gonial angle (°)	134.34	136.24	151.85	130.0 ± 6
Anterior cranial base (mm)	57.58	60.77	59.10	71 ± 3
Posterior cranial base (mm)	26.16	28.19	25.61	32 ± 3
Ramus height (mm)	47.75	43.58	40.18	44 ± 5
Anterior facial height (mm)	117.26	111.18	100.02	112.5 ± 7.5
Posterior facial height (mm)	66.07	69.08	54.87	77.5 ± 7.5
Jarabak ratio (%)	56.34	62.13	54.86	63.51 ± 1.5

SD = standard deviation.

In conclusion, maxillary DO can sufficiently advance the maxilla in CLP patients. In clinical practice, however, it is important to be aware of the complications of TMJ dislocations and reposition the joint as soon as dislocation is detected.

## Acknowledgments

The authors thank the participating patients and their families, as well as Editage (www.editage.com) for English language editing.

## Author contributions

**Conceptualization:** Tao Song.

**Data curation:** Junya Zhai.

**Formal analysis:** Binqing Wang, Junya Zhai.

**Investigation:** Yilue Zheng, Haizhou Tong.

**Supervision:** Ningbei Yin, Tao Song.

**Validation:** Yang Lü.

**Writing – original draft:** Binqing Wang.

**Writing – review & editing:** Binqing Wang, Zhewei Chen, Tao Song.

## Abbreviations

Abbreviations: Ba = basion, CLP = cleft lip and palate, CT = computed tomography, DO = distraction osteogenesis, Go = gonion, RED = rigid external distraction, TMJ = temporomandibular joint.
